# 

**Published:** 1912-03-23

**Authors:** 


### Buildings Contemplated and in Progress.

Rochdale Infirmary Extension.—The extensions to the Rochdale Infirmary are now developing and are estimated to cost close on £18,000, exclusive of laundry fittings and furnishing. The new works include a pavilion to contain some fifty-six beds and administrative and sanitary blocks. Mr. Hugh Healey, A.R.I.B.A., of Manchester, is the architect.

### HOSPITALS AND SMOKE ABATEMENT.

To-day (Saturday) an exhibition opens at the Agricultural Hall under the auspices of the Coal Smoke Abatement Society. All hospital officials anxious to determine the value and practicability of the latest appliances for the use of smokeless fuels can see them at this exhibition. There will also be a large display of gas-cooking appliances, for hot-water supply and furnace ware. Tickets can be obtained from the Gas Light and Coke Company by any consumer who applies at their offices.

### Florence Nightingale Memorial.

The following contributions have been received since last week by Mr. G. Q. Roberts: His Grace the Duke of Westminster, £50; Sir John Grinlinton, £10; Septimus Vaughan Morgan, Esq., £5 5s.; Officers' Mess 4th Batt. Royal Fusiliers, £5 5s.; Major-General Luke O'Connor, £5 5s.; Mrs. Henry Lambton, £5; Captain William Lambton, £5; Her Grace the Duchess of Bedford, £5; Clara Lady Henley, £5; Colonel S. G. Stopford Sackville, £5; George A. Macmillan, Esq., £5; the Western Cavalry Depôt, Seaforth, £4 10s.; Miss D. E. Cordingley, £4; E. Vaughan Morgan, Esq., £3 3s.; T. P. Warren, Esq., £3 3s.; Officers and Men, Military Hospital, Brighton, £2 7s. 6d.; J. H. Buxton, Esq., £2 2s.; County School for Girls, Tonbridge, £2; Staff of 3rd Southern General Hospital, Oxford, £1 12s.; 11th Field Company Royal Engineers, £1 7s. 6d.; the Officers, the Depôt, Renmore Barracks, Galway, £1 5s.; Medical Officers Royal Herbert Hospital, Woolwich, £1 2s. 6d.; Lady Guillum Scott, £1 1s.; Officers 2nd Batt. Royal Inniskilling Fusiliers, £1 1s.; Sir Ernest George, A.R.A. (statue), £1 1s.; S. H. Romilly, Esq., £1 1s.; Edward B. Merriman, Esq., £1 1s.; Captain Luscombe, £1 1s.; Right Hon. James Round, £1 1s.; Mrs. Ernest Blunt (in addition to £1 1s. already received), £1 1s.; W. F. Lawrence, Esq., J.P., £1 1s.; M. W., £1 1s.; J. B. Hilditch, Esq., £1 1s.; Major-General G. C. Rowcroft, £1 1s.; Mrs. Holland, £1 1s.; F. E. Hesse, Esq., £1 1s.; Edmund J. James, Esq., £1 1s.; Hon. Mrs. Arthur Henniker, £1 1s.; Lieutenant-Colonel G. S. Haines, £1; St. Thomas's Church, Seaforth, 10s. 7d.; Miss Fawcett, 10s.; Miss Ida M. Lea (annuities), 10s.

